# Understanding of the transition to adult healthcare services among individuals with VACTERL association in Sweden: A qualitative study

**DOI:** 10.1371/journal.pone.0269163

**Published:** 2022-05-27

**Authors:** Ann-Marie Kassa, Gunn Engvall, Michaela Dellenmark Blom, Helene Engstrand Lilja

**Affiliations:** 1 Department of Women’s and Children’s Health, Uppsala University, Uppsala, Sweden; 2 Department of Paediatric Surgery, University Children’s Hospital, Uppsala, Sweden; 3 Department of Pediatrics, Institute of Clinical Sciences, Sahlgrenska Academy, University of Gothenburg, Gothenburg, Sweden; 4 Department of Paediatric Surgery, The Queen Silvia Children’s Hospital SU/Östra, Gothenburg, Sweden; Shandong Public Health Clinical Center: Shandong Provincial Chest Hospital, CHINA

## Abstract

Current knowledge of transitional care from the perspective of individuals with congenital malformations is scarce. Their viewpoints are required for the development of follow-up programs and transitional care corresponding to patients’ needs. The study aimed to describe expectations, concerns, and experiences in conjunction with transfer to adult health care among adolescents, young adults, and adults with VACTERL association, (i.e. vertebral defects, anorectal malformations (ARM), cardiac defects (CHD), esophageal atresia (EA), renal, and limb abnormalities). Semi-structured telephone interviews were performed and analyzed with qualitative content analysis. Of 47 invited individuals, 22 participated (12 males and 10 females). An overarching theme emerged: Leaving the safe nest of pediatric health care for an unfamiliar and uncertain follow up yet growing in responsibility and appreciating the adult health care. The participants described expectations of qualified adult health care but also concerns about the process and transfer to an unfamiliar setting. Individuals who were transferred described implemented or absence of preparations. Positive and negative experiences of adult health care were recounted including being treated as adults. The informants described increasing involvement in health care but were still supported by their parents. Ongoing follow up of health conditions was recounted but also uncertainty around the continuation, missing follow up and limited knowledge of how to contact health care. The participants recommended information ahead of transfer and expressed wishes for continued health care with regular follow up and accessibility to a contact person. Based on the participants’ perspective, a transitional plan is required including early information about transfer and follow up to prepare the adolescents and reduce uncertainty concerning future health care. Meetings with the pediatric and adult team together with the patient and the parents are essential before transfer. Follow up should be centralized to centers with multi-professional teams well-experienced with the condition. Further studies are warranted to evaluate the transition process for adolescents and young adults with complex congenital health conditions.

## Introduction

VACTERL association includes at least three of the following congenital malformations: vertebral defects, anorectal malformations (ARM), cardiac defects (CHD), esophageal atresia (EA), renal anomalies, and limb abnormalities [1]. The condition is rare with estimated birth prevalence in Europe 6.25/100,000 [2]. Most children require surgery during the first days of life and often repeated procedures under anesthesia during childhood [1]. Nevertheless, mortality has decreased in the recent decades due to improvements in neonatal intensive care, anesthesia, and surgical techniques [1, 3]. Potential physical sequelae such as scoliosis, bowel dysfunction, dysphagia, gastro-esophageal reflux, airway morbidity, or reduced cardiac, renal, or limb function [1, 4, 5] may impair health-related quality of life and require long term follow up [6–8], as recommended for ARM and EA by international consensus [9, 10].

During adolescence with its physical, cognitive, and psychosocial development [11], behavioral patterns affecting future adult health, such as eating and physical activity habits are established [11, 12]. For individuals with chronic conditions health status may deteriorate during this period [13] and patients with congenital malformations without regular follow up in adult life may risk adverse outcomes [14].

Already in 1993, the Society of Adolescent Medicine defined transition as “*the purposeful*, *planned movement of adolescents and young adults with chronic physical and medical conditions from child-centered to adult-oriented health-care systems”* [15]. In the literature this process is differentiated with respect to the concepts of transition and transfer. Transition of care could be described as an educational process preparing adolescents with chronic health conditions for taking responsibility for their health and lives, while the word transfer refers to the event of moving their health care [16] and medical information from pediatric to adult services [17]. Preparation for transfer should start through early information [18], the process of transition should be initiated at the age of 12–13 [10, 19] while the actual transfer should take place after completed growth and puberty [17], ideally at the age of 18–21 years [19]. Recommended components in the transition process include identifying a transition coordinator [10, 20], reassessment of the medical and psychosocial condition [10] and providing the adolescent with knowledge and skills about their medical condition [10, 20]. Furthermore, the adolescents should be encouraged in involvement and self-efficacy by seeing the doctor without their parents [10]. A well-developed transition program might entail adequate management of chronic health conditions, early discovery and treatment of complications and reduced physical and psychosocial morbidity [10]. Still, there are no guidelines for transition and transfer of patients with VACTERL association.

Experiences of transition and transfer to adult health care have been studied among patients with various chronic health conditions [21–26], but for individuals with complex congenital malformations corresponding knowledge is scarce. In recent years, the European Commission formed the European Reference Networks to strengthen the delivery of good health care and treatment for people living with a rare disease [27], which further illustrates the urgency of increasing knowledge of transitional care in people living with VACTERL association.

In this study a wide age group is included since it is valuable to identify necessary issues to be addressed in a proper transition program, both the expectations and worries among the adolescents and the experiences of an implemented transfer or the absence of transfer. Even though the oldest participants would have been transferred long ago we could learn from their experiences and wishes for transfer and follow up.

The aim was to explore the understanding of the transition to adult healthcare services among individuals with the VACTERL association.

## Material and methods

### Participants and setting

Adolescents aged 15–17, young adults (YA) aged 18–20, and adults >20–35 years with VACTERL association were recruited from three out of four Swedish pediatric surgical centers between 2016 and 2021. Eligible individuals were sent an invitation to participate in the study while two YA contacted the researchers after information through the VACTERL-specified peer organization. Table 1 shows the number of invited individuals, participants, and the median ages in the different age groups. Of 47 invited individuals 22 (12 males and 10 females) participated in the study. The pediatric surgical care in Sweden is usually completed for patients between 15 and 18 years of age, depending on hospital and diagnosis. There is no standardized transfer or transition program for individuals with VACTERL diagnosis. For children and adolescents with ARM, EA, and CHD there are nationwide follow-up programs established where the last follow up at a pediatric surgery or a pediatric cardiology clinic takes place at the age of 15. In the absence of national transition programs the existence of transfer and further follow up depends on the routines of each hospital. Adolescents with ARM, EA, and CHD may be transferred between 15 and 18 years of age.

**Table 1:** Invited individuals and participants per age group.

Group	Age group	Invited (M/F)	Participants (M/F)	Age Md (min-max)	Experience of adult health-care services
Adolescents	15–17	18 (12/6)	10 (7/3)	15.0 (15–17)	1
YA^a^	18–20	18 (8/10)	8 (4/4)	18.5 (18–20)	5
Adults	>20–35	11 (8/3)	4 (1/3)	31.5 (28–35)	4

^a^ YA = Young adults

### Method and data collection

The interviews were performed by telephone by the first author who was not involved in the regular treatment of the patients. A semi-structured interview guide, constructed by the authors, was used to cover the aim of the study. The open-ended questions concerned thoughts and expectations ahead of transfer and furthermore, experiences of the transfer process and of adult healthcare services. Follow-up questions were used to expand the answers. The interview guide is provided in supporting information in original language in S1 and S3 Files and in English in S2 and S4 Files. The interviews were audio recorded and transcribed verbatim by a professional writing agency. Reflection notes were taken after the interviews.

### Data analysis

For the analysis, qualitative content analysis with an inductive approach [28] was used as described by Graneheim & Lundman [29, 30]. After repeatedly listening and reading through the interviews while checking the transcriptions, meaning units consisting of a part of a sentence or a whole sentence or more corresponding to the aim were identified in one interview at a time. These meaning units were condensed that is, were shortened while keeping its content and were thereafter given a code such as “expectations”. Through the process of comparing, grouping, and abstracting the codes, subcategories, and categories were formulated where the content in each category shared commonality while disparities were excluded. Finally, a descriptive theme was formulated illustrating the content consistent throughout in the interviews. The software NVivo for Windows, Release 1.4.1 (QSR International Pty Ltd, Victoria, Australia) was used to organize and visualize the material.

### Ethical considerations

An invitation letter with study information and a consent form was sent out to eligible individuals and guardians. Translated information letters are provided in supporting information S6–S8 Files. Ahead of the interviews written informed consent was obtained by mail from the participants and for those below 18 years of age also from their guardians. The study was approved by the Regional Ethical Review Board in Uppsala, registration number 2015/264, (amendments 2015/264/1, 2015/264/3) and the Swedish Ethical Review Authority amendments 2019–00139 and 2020–06401. To avoid recognition of participants the quotations were only labeled with consecutive numbers and age group.

## Results

Information about the health conditions of the participants is based on what they reported in the interviews. Hence, they might not be aware of all health conditions they were born with and may not remember all surgeries they have passed. All participants were born with multiple malformations that required surgery during the neonatal period with further surgery and regular follow up during childhood. One participant among the adolescents, five YA, and all four adults had experiences of adult health care even though a formal transfer was not always described.

Median length of the interviews was 33 (17‒60) minutes; among adolescents: 31.5 (23‒42), YA: 31 (17‒39), and adults: 49 (40‒60) minutes.

The participants’ expectations, concerns, and experiences in conjunction with transfer to adult health care are described in six categories: Imagining transfer, Experiences of transfer, Perceived outcome of transfer, Gradually growing in responsibility but still supported by parents, Describing follow up, Expectations and wishes for future good health care (Table 2). The content is described in 15 subcategories and illustrated by quotations followed by a number and the age group for each participant. An overarching theme was formulated: Leaving the safe nest of pediatric health care for an unfamiliar and uncertain follow up yet growing in responsibility and appreciating the adult health care.

**Table 2:** Overarching theme, categories, and subcategories describing expectations, concerns, and experiences in conjunction with transfer to adult health care.

Overarching theme	
*Leaving the safe nest of pediatric health care for an unfamiliar and uncertain follow up yet growing in responsibility and appreciating the adult health care*.	
Categories	Subcategories
**Imagining transfer**	More or less expectations
	Uncertainty, sadness, and various concerns
**Experiences of transfer**	Varying preparation ahead of transfer
	Process of transfer and special arrangements
	Transfer not accomplished
**Perceived outcome of transfer**	Mixed experiences of adult health care
	Treated differently in adult health care but feeling grown up
**Gradually growing in responsibility but still supported by parents**	Increasing participation in treatment and care supported by parents
	Increasing independence but accompanied by parents
**Describing follow up**	Ongoing follow up
	Uncertainty or lack of follow up
	Limited knowledge of access to health care
**Expectations and wishes for future good health care**	Conveyed wishes and recommendations for transfer process
	Continued follow up and good care
	Contact person desired

### Imagining transfer

The adolescents and the YA described their expectations, concerns, and emotions ahead of transfer to adult health care.

#### More or less expectations

Adolescents and YA, recounted expectations of professional health care in the adult care unit as being as good as in the children’s. The adolescents described that they expected to meet healthcare professionals (HCP) with experience and competence. *“I wish I could come to a place where they know something about esophageal atresia and asthma and all that”* (1 adolescent). Furthermore, the YA anticipated that the HCP in the adult care unit would have more experience of grown-ups with similar health conditions and that they would describe other patient’s experiences of the health condition.

More information both practical and about the health condition was expected by the adolescents as well as nice reception and atmosphere. They further regarded the transfer as a natural step entailing increased involvement in their health care through increased expectations regarding their personal responsibility and through more direct communication with HCP. Other informants from all age groups described that they had not thought about what advantages transfer would bring.

#### Uncertainty, sadness, and various concerns

Some adolescents expressed uncertainty since they did not know what the transfer would imply nor the timing nor where they would be transferred. While some participants from all age groups did not worry at all ahead of transfer other adolescents and YA described sadness at leaving the familiar HCP in the children’s care unit and concerns about losing the security by being transferred to a new clinic with unacquainted HCP. The adolescents expressed worries about changing to less knowledgeable HCP, when follow up was planned to be performed in a primary healthcare center. *“I don’t think I’m really that worried*, *but it’s probably mostly if I were to switch to a health center*, *for example … where they don’t know anything about esophageal atresia*, *so I’m afraid*, *yes*, *that they don’t have the knowledge”* (1 adolescent). There was also concern among the YA that their future care would be less thorough since the HCP would put more responsibility on the patient.

### Experiences of transfer

The informants described if and how the transfer process was performed and the presence or absence of information and special arrangements in conjunction with it.

#### Varying preparation ahead of transfer

Informants from the various age groups described what information they had been given about a forthcoming transfer conveyed by the doctor at a hospital visit or by their parents. Others described that they only received a letter with an appointment to the adult clinic for an information visit. *“No*, *so I just got the letter and then I don’t know if … maybe my mother had talked to … to someone else on the phone*. *But I hadn’t talked to them”* (2 adolescent). *YA* described that they were well prepared since the information was given in good time and in some cases both in writing and orally. Conversely, other adolescents and YA could not recall any information and some YA did not know if the present follow up was performed in children’s or adult’s clinic. According to the adolescents, preparations through discussions about the transfer within the family were limited.

#### Process of transfer and special arrangements

The YA who were transferred described that the transfer took place between 15 and 18 years of age according their types of malformations. Most of them described that after receiving information they got an appointment to the new adult service. A few informants described special arrangements made for the transfer, such as meeting HCP from adult services in advance or a transfer visit together with their parents, meeting both the pediatric and adult doctors during which the medical information was conveyed from the former to the new specialist. *“It felt good*, *then my … former doctor could talk about … what has been done and how everything had gone and how things are and so on*. *Because then the others can … get a good picture and they have*, *yes*, *close contact … they work together a little … my mother was there too … I thought that was great”* (3 YA). Others described that the pediatric doctor responsible chose, together with the parents, to transfer the adolescent to a specialist colleague they knew well.

#### Transfer not accomplished

Most of the adolescents had not been transferred to adult health care and some of the YA had still not visited any adult clinic. The adult participants described transfer to specialists such as neurologists, hand surgery, or plastic surgery specialists but no proper transfer for EA and ARM from pediatric surgery to corresponding adult services. Still, they all had some experience of adult healthcare service. *(About when I stopped attending the pediatric clinic)”… what was said then was that if there were any further problems … we would have continued contact*, *but since it has all worked so well for me*, *there’s not been a need … I haven’t had to have any further operations … yes*, *it has sort of just … ebbed away*, *you could say”* (4 adult).

### Perceived outcome of transfer

The adolescents, YA, and adults described their perceptions of the outcome of transfer including both positive and less positive experiences of adult health care.

#### Mixed experiences of adult health care

Those YA and adults who had been transferred to adult health care described it as a positive experience. They spoke of a good and professional reception from the HCP in the adult department and the YA expressed that they were even positively surprised by the adult health care. Also, one adolescent described that visiting the adult department felt like an advantage, that it brought a feeling of being more grown up. Furthermore, the YA described a sense of safety with access to support for their current medical needs. “*… I thought that*… *it went well … I ended up in safe hands and I felt safe already early on*… *and I have not had … any reason to feel insecure since then”* (5 YA).

In contrast, the adolescent who was transferred recounted a negative experience at their first visit to the adult clinic, not being informed and prepared for an examination of their anal region. *“I remember that I was there … then I thought I was just there for questions and so on but then they wanted to look at like the scar like … on my butt … and then I would have thought I could have gotten information about that beforehand”* (2 adolescent). Also the adults described some negative experiences of the adult care when their suggestions were not accepted or when they were nonchalantly received and forced to argue for the right to receive care in the event of acute sickness.

#### Treated differently in adult health care but feeling grown up

Those participants who had experienced adult health care described the services as comparable to pediatric services, but some differences were recounted such as another type of environment, unfamiliar HCP, and different reception of the patients. According to the informants, the pediatric HCP had focused more on creating a positive atmosphere while the adults’ HCP behaved more matter-of-factly. Furthermore, the YA and the adults described that they were treated more as adults in the way the HCP communicated with them. *“The difference is that I perceived that I was treated like a child or adolescent at that clinic and treated like an adult at the adult clinic … how the doctors formulate themselves when they talk to me and how serious they are and how … they say things because they expect that I understand more now than I did as a child”* (6 adult).

### Gradually growing in responsibility but still supported by parents

The informants described their involvement in their treatment and care through increasing participation, responsibility, and independence but still seeking support from their parents.

#### Increasing participation in treatment and care supported by parents

Adolescents and YA described that they were involved in the discussions about their health care, but that there was not much they could decide about. Since they did not have enough knowledge, they left the decisions about their treatment to the HCP. Other adolescents described that they were allowed to participate and to some extent influence decisions about treatment and practical arrangements. They also recounted how they spoke up when not being comfortable with the arrangements. Some YA and adults described how they participated in decisions and for instance had an impact on changing what doctor was responsible or as teenager taking a decision about surgery said: *“Yes*, *I have been involved so much … yes*, *all the time*, *been involved and decided”* (7 YA). Some adults recounted that they wanted to increase their knowledge of their disease to enhance their possibilities to influence treatment.

Informants from all age groups described how their parents had previously been involved in their care by obtaining information and influencing treatment and according to the adolescents and YA they still were involved. Furthermore, adolescents expressed how they expected that their parents would hinder any inappropriate treatment. *“I have always assumed that if they offer a treatment that would not be good for me*, *she (mother) would intervene”* (8 adolescent).

The adolescents described that it was the parents who initiated contact with the health services when necessary while the YA described increasing responsibility for creating contacts or making appointments. *“*… *it is much more your own responsibility*… *and book your own times and keep track of that and so on*, *so it becomes a different way of doing things*” (5 YA). However, in many cases the parents were still responsible for contacting health care.

#### Increasing independence but accompanied by parents

The adolescents described how their parents or a relative accompanied them on hospital visits and supported them during hospitalizations and YA described how they felt safe thanks to their parents’ company in hospital. Most of the YA recounted that they were still now accompanied by their parents. *“… If I need to go for checks and suchlike … she always comes with me … because she has always been with me … since I was little*, *so it’s nice that she … is with me”* (9 YA). However, a few adolescents and YA spoke of visits to the hospital without their parents and described these as good experiences. Even some of the adults described how the parents had supported them in getting care during acute illness.

### Describing follow up

Some informants described ongoing follow up of their health conditions but also uncertainty about the continuation. Others described the lack of follow up and limited knowledge regarding how to contact health care.

#### Ongoing follow up

The adolescents and most of the YA described current follow up with continuity in diverse specialist clinics, some of them were still performed in pediatric services and some in adult health services. *“There are some*… *annual check-ups at both places … if I*… *have something I need to bring up*, *I get in touch*. *But usually only*… *once a year … at the different places”* (5 YA). Among the adults, some of the participants had through their own initiative gained access to late specialist follow up. Some of the YA and the adults who were followed by a general practitioner at a health center reported difficulties such as lack of knowledge of their particular diagnosis, as well as a general lack of time and continuity among the doctors.

#### Uncertainty or lack of follow up

The adolescents expressed uncertainty since they were not informed whether there would be continued check-ups, nor where and how these would be implemented or organized in the future. *(About continued contact with the health care) “it can be a little annoying not knowing*, *but if they have not planned something then it can’t be helped”* (10 adolescent). Other YA reported that the follow up for certain malformations such as EA was not planned or completed by 11 to 13 years of age. Furthermore, the adults recounted that there was currently no regularly follow up of the VACTERL conditions while there was for other medical conditions such as asthma, lung problems, and epilepsy. *“And then*… *they kind of let go of this with VACTERL*… *then it’s become very*, *very focused on … I have asthma"* (11 adult).

#### Limited knowledge of access to health care

Within all age groups there were participants who lacked contact persons and did not know whom to turn to concerning their health condition. However, both adolescents and YA recounted that their parents knew whom to contact. *(About whom to contact if problems) “No*, *I actually don’t know*, *I thought it was the same as I had before*, *I mean the same doctor*, *but … because mom has contact with one*. *But I actually don’t know right now who I should talk to”* (12 YA).

### Expectations and wishes for future good health care

The participants expressed their recommendations for the transfer process through information and meeting the adult’s HCP ahead of transfer. Furthermore, they described their expectations and wishes of continued good health care with regular follow ups and accessibility to a contact person.

#### Conveyed wishes and recommendations for the transfer process

The adolescents conveyed wishes that they would receive proper written and oral information about the transfer process and about their future HCP *“… Before they change*, *I think … that they should*, *as it were*, *properly inform you that you are now moving from child to adult … I don’t go to so many check-ups anymore so*, *well then maybe it would have been relevant to send me a letter or an email*. *But … if you get check-ups every month*, *then maybe it’s better to just talk about it … when you turn eighteen*, *you’ll be moving to an adult clinic”* (13 adolescent). The YA and adults recommended that both oral and written information should be provided at least six months before the transfer took place. The adolescents wanted a slow process and also expressed a wish that their medical information should be transferred to the adult unit.

The adolescents did not feel it was necessary to meet the new HCP in advance but the YA did on the contrary suggest an arranged meeting with a multi-professional team from the adult clinic to create a sense of security and recognition of the actual transfer.

Furthermore, YA and adults recommended one of more joint meetings together with the pediatric and the adult specialists for information transfer. *“So … the new doctor is in on a meeting with the old doctor so maybe he can learn … a little about what has worked for that particular individual”* (5 YA). Another wish conveyed by YA was the possibility to influence the choice of the future adult doctor.

#### Continued follow up and good care

For the future, informants from all age groups expressed their wishes for continued regular check-ups to follow the progress of their health condition. *“I would like … yes serious check-ups like those I have had so far to make sure that no issues arise for me*. *Yes*, *and the ability to fix the problems should they arise”* (8 adolescent). The adolescents expected immediate medical help and support in the case of deterioration of their condition and the YA wished for continued care that was as good as before and that the various follow-up visits should, as much as possible, be located to one center.

#### Contact person desired

The informants in all age groups wanted information regarding who to contact when necessary and the YA and the adults suggested that the patients should be offered a contact person who could forward questions and establish a contact with a specialist doctor.*”… You could be offered … a contact person*, *who you can turn to … if there is something I have a question about*, *you can contact them … they may not themselves be able to answer all the questions*, *but then they can find out … then you can talk to this doctor … but just to clearly have someone to turn to”* (4 adult).

## Discussion

To the best of our knowledge this is the first study reporting on expectations, concerns, and experiences in conjunction with transfer to adult care among adolescents, YA, and adults with VACTERL association. Knowledge of expectations and experiences of the patients is necessary to develop follow-up programs and transitional care that correspond to the need of the patients.

### Information ahead of transfer

Less than half of the participants in the present study had experienced actual transfer to adult health care. Most of the adolescents had not been transferred and most of them were not worried about the transfer, while others did express uncertainty, not knowing when or where to be transferred. Likewise, adolescents and YA participating in other studies have talked about a lack of information ahead of the transfer [24, 31, 32]. They might have been informed but not taken it in [24], maybe because they were not actively involved in the communication. To reduce the uncertainty and potential worries about future health care it would be appropriate to provide early information about the future planned transfer [17, 18, 33] and to actively include the adolescents in the discussions [17]. In line with a previous study of transition from pediatric to adult care in YA with type 1 diabetes [24], the adolescents in our study wanted to receive written and oral information about the transfer process and their future HCP. It has been recommended that information should be given repeatedly starting from the early teens in adolescents with chronic conditions [19].

### Expectation of information about the health condition

Some adolescents in our study recounted expectations for more information about their diagnosis when transferred. Participants in other studies have witnessed limited knowledge of their malformation diagnosis among adult’s HCP [26, 34]. Since the pediatric surgeons have the greatest experiences of the malformations included in the VACTERL association this information should be provided before transfer. The adolescents and YA should be provided with age-adapted written information about their medical background and present health condition [10].

### Experiences of adult health care

Despite concerns and worries about leaving the pediatric care, experiences of adult health care were mainly described as positive in our study. Some participants even described that they were positively surprised at the good reception and care which they experienced as being as good as in the pediatric service. One advantage they described was that of being treated in a more grown up manner in the way the HCP communicated. As reported by other adolescents and YA in a previous study the direct communication with the HCP and increased responsibility could be experienced as a culture shock [21] but at the same time be seen as a positive natural step towards adulthood [35].

In comparison however, some individuals described negative incidents such as lack of information about the content of a hospital visit and situations creating feelings of being disregarded during acute illness and procedures. Furthermore, the participants recounted disparities between the services, describing how the adult’s HCP behaved more matter-of-factly while the pediatric HCP were more relationship focused. Similarly, other YA and adults with long-term health conditions have described the HCP in adult health care as less caring [23], less personal [24], disease-focused and not as easy to form relationship with [35] compared to the HCP in the pediatric services.

### Need of specialized follow up

The adults in our study described the absence of follow up and that they had to take their own initiative to seek out medical help for their symptoms. Furthermore, several of the participants did not know whom to contact in the case of health problems related to their malformations. Other studies have also pointed out similar problems for patients with for example colorectal malformations who were dissatisfied with their follow up after leaving the pediatric clinic [6] and were without knowledge of where to seek support for medical problems connected to their diagnosis [26, 36].

There are to our knowledge no comprehensive program for follow up and transfer of individuals with the diagnosis of VACTERL association. For the diagnoses ARM, EA, and CHD nationwide follow-up programs exist in Sweden with the last follow up at the age at 15, but still transition programs are missing. Recommendations for transition of care for patients with ARM to adult services have been provided by the ARM-net consortium [10] and international networks have agreed on the necessity of structured life-long interdisciplinary follow up [9] with detailed guidelines for patients with EA [7]. However, there are difficulties associated with finding appropriate qualified healthcare services for the follow up of adult patients with congenital malformations. Participants in our study who were directed to follow up through a General Practitioner at their primary health center described concerns about meeting HCP less knowledgeable about their diagnosis. They described lack of knowledge, time, and continuity among the doctors in these healthcare facilities. Furthermore, patients with colorectal malformations have reported that surgeons specialized in adults lack sufficient knowledge about the diagnosis [34] and they have also described adult care providers as ignorant of the disease and its potential complications [26].

One way to improve the follow up of adult patients with congenital malformations could be by using a model implemented in Singapore. Patients with ARM are followed in a specialized clinic including both a colorectal surgeon associated to the adult clinic and a pediatric surgeon with experience of children with ARM [37]. The ARM-net consortium suggests that the pediatric surgeons should promote the presence of adult specialists during the repair of anorectal defects in neonates and any other surgical procedures in childhood [10]. Furthermore, knowledge about the patient’s condition could be improved by meetings with the pediatric and adult team together with the patient and parents as suggested by the participants in the present study and also recommended by the ARM-net consortium [10]. These meetings could result not only in the transfer of knowledge but also in an increased sense of security for the patient by meeting the adult specialist and witnessing what information is transferred.

The participants in our study who described a lack of follow up were predominantly the adults. Since the awareness of the importance of continued follow up into adulthood has increased, hopefully all adolescents with congenital malformations will obtain regular follow ups. However, according to our findings regarding deficient and absence of follow up, such a program for VACTERL association including an elaborated transition plan is needed. As recommended in the literature, the transition should start in the early teens by informing the adolescent about the plan for future follow up [19]. Furthermore, their knowledge about their diagnosis [10, 20], their self-efficacy, and their responsibility for their treatment could gradually be increased by involving them in the discussions and seeing the HCP without parents present [10]. In line with the participants’ conveyed wishes, it is also of great importance to appoint a contact person with access to the specialists in the different areas to connect the patients to these essential caregivers.

### Trustworthiness

We have addressed trustworthiness of the study according to the criteria of Lincoln and Guba, as described by Polit and Beck [38]. To enhance credibility we recruited individuals from three pediatric surgical centers to obtain a larger and mixed sample. Furthermore, the interviews were performed by the researcher who was not involved in the regular care of the participants and had completed post-graduate courses in interview techniques and qualitative content analysis. During the analysis process with inductive approach the interviews were repeatedly read before identifying meaning units corresponding to the aim. Measures were taken to stay close to the transcribed interviews by checking the codes and categories through frequently moving back to the transcriptions. All data relevant to the aim were included and sorted into the categories. Furthermore, variation in aspects were described and representative quotations chosen from several participants. Dependability refers to consistency of the data collection and analysis over time. To enhance stability in the data collection the interviews were performed by one researcher staying close to the interview guide. Confirmability referring to the objectivity of the analysis was enhanced through the cooperation between the co-authors during the analysis process to reduce the risk of subjective interpretations. To minimize the errors in the analysis and to increase credibility, dependability and, confirmability the codes were reviewed and categories and subcategories were discussed together with the co-authors until consensus was reached. Transferability to other contexts and groups may be affected by the small study group and the limited data presented on clinical characteristics of the participants. Taken together this might make our results less transferable to other groups with long-term health conditions, but it still provides valuable viewpoints from the patients on the transfer process. Nevertheless, what could strengthen transferability to similar settings is comparable experiences described in the literature and that the participants were recruited from more than one pediatric hospital.

### Limitations

All interviews were performed by telephone. The wordless communication may be lost but still we assess that the advantages prevail. The participants may find it easier to talk on the telephone rather than face to face with an unknown researcher. Furthermore, telephone interviews might be a prerequisite for recruiting informants in these age groups.

In the present study we decided to use the designation “transfer”. A proper transition process includes much more than just shifting from pediatric to adult clinics and involves training to make the adolescents and YA ready to take responsibility for their health and treatment. Such a process might not have been in place for our informants and probably it would have been more difficult for the patients to identify and describe.

The information about the health conditions of the participants is based on what they told in the interviews and they might not be aware of all health conditions they were born with and may not remember all surgeries they have went through.

Less than half of the invited individuals participated which may represent a selection bias. However, our material includes both positive and negative descriptions. Due to the recruitment difficulties we choose to include persons above the age of 20. Among them four out of 11 invited participated, which limits representativeness for this age group. Nevertheless, they conveyed valuable information about conditions for older VACTERL patients.

## Conclusions

This study has made visible expectations, concerns, and wishes for transfer and future follow up among adolescents and adults with complex congenital anomalies. The individuals have provided valuable experiences which should be taken into account when developing a transition program for individuals with VACTERL association. From the perspective of adolescents, YA, and adults with VACTERL association, we conclude that the transfer to adult health care could be improved. A transitional plan is required including early information about transfer and follow up to prepare the adolescents and reduce uncertainty concerning future health care. Before transfer, meetings with the pediatric and adult team together with the patient and their parents are essential and follow ups should be centralized to centers with multi-professional teams well-experienced with the condition. The patients should be provided with information about an appointed contact person to turn to when needed. Further studies are warranted to evaluate the transition process for adolescents and young adults with complex congenital health conditions.

## Supporting information

S1 File Interview guide for adolescents in original language (Swedish). (PDF)

S2 File Interview guide for adolescents translated into English. (PDF)

S3 File Interview guide for young adults and adults in original language (Swedish). (PDF)

S4 File Interview guide for young adults and adults translated into English. (PDF)

S5 File COREQ checklist. (PDF)

S6 File Information letter adolescents. (PDF)

S7 File Information letter guardians. (PDF)

S8 File Information letter young adults and adults. (PDF)
