# Supplementary material for: Understanding of the transition to adult healthcare services among individuals with VACTERL association in Sweden: A qualitative study
Source: PLoS One. 2022 May 27;17(5):e0269163. doi: 10.1371/journal.pone.0269163 (PMC9140225; doi:10.1371/journal.pone.0269163)
Supplement: S1 File — (PDF) [file pone.0269163.s001.pdf]

S1 File. Interview guide for adolescents in original language (Swedish).

Inledande frågor/ information

Presentation av mig själv, varför intresserad.

Syfte med intervjun: Upplevelser av vården och hur du önskar att den ska fungera

Hur går det till?

Berätta gärna fritt!

Inga svar är rätt eller fel det är dina erfarenheter och upplevelser och tankar det handlar om.

Jag spelar in - skriver ner intervjun. Intervjun enbart märkt med en sifferkod.

Kodlista som enbart jag och mina handledare har tillgång till.

Anonym sammanställning av resultatet. Ingen vet vad just du svarat.

Frivilligt!

Har du några frågor innan vi börjar intervjun?

Warming up talk

Vad gör du till vardags? Vilken skola går du i, vilken klass? Vad är ditt favoritämne?

Vad tycker du om att göra på fritiden?

Vad tänker du dig att läsa sedan?

Allmänt om sjukhus och sjukhusvistelser

Har du varit mycket på sjukhus?

Vilka olika sjukhus har du varit på?

Vill du berätta vilka problem du hade när du föddes?

När du tänker tillbaka på alla gånger du varit på sjukhus:

Kommer du ihåg några speciella tillfällen? Kan du berätta mer om det?

Några speciella händelser? Kan du berätta mer om det?

Vad är det som har varit bra när du varit på sjukhus?

Vad är det som varit mindre bra eller dåligt på sjukhus?

Berätta om vad du har tyckt varit jobbigt eller obehagligt

Kan du berätta hur du upplevde det?

Berätta när du har känt dig rädd

Berätta om när du upplevt smärta

Hur tycker du att personalen har varit mot dig?

Vad har varit bra i kontakten med personalen? Vad har varit mindre bra?

Någon speciell händelse som du minns? Positivt eller negativt?

Om du försöker att tänka på alla tillfällen du varit på sjukhus vad känner du då?

Är det något mer du vill berätta om upplevelser på sjukhus?

Nuläge

Hur ofta är du på sjukhuset nu för tiden?

Vilken typ av mottagning, avdelning? (vuxen eller barn?)

Vad är det som gör att du behöver komma till sjukhuset?

Brukar dina föräldrar följa med dig?

Vem tar kontakt med sjukhuset när det är problem?

Hur mycket får du vara med och bestämma om din behandling?

Nu ska vi gå över och prata om hur det kan bli med kontakterna med sjukvården i fortsättningen när du blir äldre

Tre alternativa fortsättningar av intervjun:

1. Planerad fortsatt uppföljning

Information om överföring

Tror du att du kommer att behöva fortsätta att komma till sjukhuset när du blivit äldre? 18 år?

Vet du hur det blir i fortsättningen? Kommer du att få byta avdelning/mottagning?

Vet du vilken klinik eller mottagning som du ska komma till när du blir 18 år?

Har de pratat om det på "din" mottagning? Har ni pratat om det länge?

Har ni pratat om det i familjen?

Förväntningar och farhågor inför överföring

Om det är så att du måste byta mottagning/avdelning: Vad tänker du om det? Hur tror du att det blir?

Hur tänker du om att lämna den mottagning/avdelning du varit på?

Känner du något speciellt inför det? Hur känns det?

Hur tänker du om att byta den personal som tar hand om dig nu?

Vilka förväntningar har du inför att komma till en ny mottagning/avdelning?

Vad tror du kommer att bli bra med att byta avdelning?

Vad önskar du dig från den mottagning där du sedan får fortsätta att gå?

Är det något du är orolig för i det här att byta avdelning?

Hur önskar du att kontakten med sjukvården kommer att fungera i fortsättningen när du fyllt 18 år?

2. Ingen planerad fortsatt uppföljning

Hur tänker du om att det inte finns någon planerad uppföljning?

Vad sa dom på barnmottagningen när du avslutades?

Fick nu någon information från barnmottagningen om fortsatt uppföljning när du avslutades där?

Vet du vart du ska vända dig om du får problem i fortsättningen?

Tror du att dina föräldrar vet det?

Hur önskar du att kontakten med sjukvården kommer att fungera i fortsättningen när du fyllt 18 år?

3. Redan överförd till vuxensjukvård

Erfarenheter av överföring alternativt att få lämna barnsjukvården

Hur länge fick du gå till barnsjukvården?

När flyttades du över till vuxensjukvården?

Kommer du ihåg vad du tänkte inför överflyttningen?

Kommer du ihåg vilka förväntningar du hade för överflyttningen?

Kommer du ihåg om du var orolig för något?

Förberedelse

Kommer du ihåg hur det förbereddes?

Fick du information i förväg? Långt i förväg? Hur fick du information? När?

Vilken typ av information?

Vem var det som var inblandad i processen?

Fick du träffa vuxenpersonalen innan du överflyttades helt? Berätta mer om det.

Erfarenheter av processen

Vad tänker du så här efteråt om överflyttningen?

Hur var det att byta klinik och personal?

Svårigheter? Nackdelar? Fördelar?

Om du jämför barnsjukvården och vuxensjukvården:

Vad är skillnaden mellan avdelningar och mottagningar för barn jämfört med för vuxna?

Vilka är likheterna?

Vad har blivit annorlunda i din kontakt med vården efter överflyttningen/efter att du blivit vuxen?

Hur tycker du det har det blivit?

Vad har blivit bättre? Vad har blivit sämre?

Praktiska kontakter

Hur kan du komma i kontakt med din avdelning eller mottagning när du behöver det?

Hur fungerar det att få kontakt?

Vem tar kontakt med vården när det behövs?

Följer dina föräldrar med?

Hur mycket är du med och bestämmer om din vård?

Vilka **önskemål h**ar du för fortsättningen i din kontakt med sjukvården?

Gemensam fortsättning av intervjun 1+2+3

Vilka råd kan du ge i samband när vi ska flytta över ungdomar till vuxensjukvården?

Förslag på hur man kan göra på ett bra sätt?

Är det något mera du vill ta upp och berätta?

Sammanfattning hur jag uppfattat informationen i intervjun

Tack för att du delade med dig!

Får jag återkomma till dig om jag har frågor?

Uppföljningsfrågor:
- Hur upplevde du det?
- Hur menar du då?
- Kan du beskriva…..?
- Kan du berätta…….?
- Hur kändes det då….?
- Vad gjorde du då?
- Kan du säga något mer om det?
