# Supplementary material for: Understanding of the transition to adult healthcare services among individuals with VACTERL association in Sweden: A qualitative study
Source: PLoS One. 2022 May 27;17(5):e0269163. doi: 10.1371/journal.pone.0269163 (PMC9140225; doi:10.1371/journal.pone.0269163)
Supplement: S2 File — (PDF) [file pone.0269163.s002.pdf]

S2 File. Interview guide for adolescents in English

Introductory questions/information

Presentation of myself, why I'm interested.

Purpose of the interview: Experiences of health care and how you would like it to function

How does it work?

Feel free to speak frankly!

There are no right or wrong answers, it is your experiences, understanding and thoughts that this is about.

I record - transcribe the interview. The interview is only marked with a number code.

A code list that only I and my supervisors have access to.

Anonymous compilation of the results. Nobody knows what specifically you have answered.

Voluntary!

Do you have any questions before we start the interview?

Warming up (talk)

Warming up talk

What do you do in your daily life? What school do you go to, which class? What is your favorite subject?

What do you like to do in your spare time?

What are you going to study next?

General information about hospitals and hospital stays

Have you been to hospital a lot?

What different hospitals have you been to?

Would you like to tell me what problems you had when you were born?

When you think back on all the times you've been in hospital:

Do you remember any special occasions? Can you tell me more about them?

Any special events? Can you tell me more about them?

What has been good about when you've been in hospital?

What has not been so good or even bad in hospital?

Tell me about what you have found difficult or unpleasant

Can you tell me how you experienced this?

Tell me about when you have felt scared

Tell me about when you experienced pain

How do you feel the staff has been towards you?

What has been good about your contact with the staff? What hasn't been so good?

Any special event you remember? Good or bad?

If you try to think of all the times you've been to hospital, how does that make you feel?

Is there anything else you want to tell us about your hospital experiences?

How often are you in hospital these days?

What type of clinic, ward? (adult or pediatric)

Why do you need to come to hospital?

Do your parents usually come with you?

Who contacts the hospital when there are problems?

How much can you be involved in deciding on your treatment?

Now we will go over to talking about how contacts with the healthcare system in the future when you get older can be

Three alternatives for the continuation of the interview:

1. Planned further follow-up

Transfer information

Do you think you will need to keep coming to the hospital as you get older? 18 years?

Do you know what it will be like in the future? Will you need to change wards/clinics?

Do you know which clinic you will need to attend when you turn 18?

Have they talked about this at "your" clinic? Have you talked about it for a long time?

Have you talked about it in your family?

Expectations and fears prior to transferring

If you have to change clinic/ward: What are your thoughts about that? How do you think it will be?

How do you feel about leaving the clinic/ward you have been to?

Do you feel anything special about it? How does it feel?

How do you feel about changing from the staff who take care of you now?

What are your expectations about coming to a new clinic/ward?

What do you think will be good about changing wards?

What do you hope for from the new clinic you will be attending?

Is there anything you are worried about when it comes to changing wards?

How would you like your contact with the healthcare system to work in the future when you've turned 18?

2. No planned further follow-up

How do you feel about the fact that there is no planned follow-up?

What did they say at the pediatric clinic when you were finished there?

Did you receive any information from the pediatric clinic about further follow-up when you were finished there?

Do you know where to turn if you have problems in the future?

Do you think your parents know?

How would you like your contact with the healthcare system to work in the future when you've turned 18?

3. Already transferred to adult health care

Experiences of transfer or of leaving pediatric care

How long did you have to attend pediatric care?

When were you transferred to adult care?

Do you remember what you thought before the transfer?

Do you remember what expectations you had concerning the transfer?

Do you remember if you were worried about anything?

Preparation

Do you remember how it was prepared?

Did you get information in advance? Far in advance? How did you get the information? When?

What kind of information?

Who was involved in the process?

Did you get to meet the adult staff before your transfer was completed? Tell me more about that.

Experiences of the process

What are your thoughts now after the transfer?

What was it like to change clinics and staff?

Difficulties? Disadvantages? Benefits?

If you compare pediatric health care and adult health care:

What is the difference between wards and clinics for children compared to those for adults?

What are the similarities?

What is different in your contact with health care after the transfer/after you have become an adult?

How do you think it has become?

What has got better? What has got worse?

Practical contacts

How can you get in touch with your ward or clinic when you need to?

How does getting in touch work?

Who contacts the healthcare services when necessary?

Do your parents come with you?

How much are you involved in deciding on your care?

What **wishes** do you have for the continuation of your contact with your health care?

Common continuation of the interviews 1 + 2 + 3

What advice would you like to give in connection with when we transfer young people to adult care?

Suggestions on how to do this in a good way?

Is there anything else you want to bring up and talk about?

Summary of how I perceived the information in the interview

Thank you for sharing this with me!

May I get back to you if I have any questions?

**Follow-up questions:**	How did you experience it?
	How do you mean?
	Can you describe…..?
	Can you tell me about …….?
	How did it feel then….?
	What did you do then?
	Can you tell me something more about that?
