# Supplementary material for: Understanding of the transition to adult healthcare services among individuals with VACTERL association in Sweden: A qualitative study
Source: PLoS One. 2022 May 27;17(5):e0269163. doi: 10.1371/journal.pone.0269163 (PMC9140225; doi:10.1371/journal.pone.0269163)
Supplement: S3 File — (PDF) [file pone.0269163.s003.pdf]

S3 File. Interview guide for young adults and adults in original language (Swedish).

Inledande frågor/ information

Presentation av mig själv, varför intresserad.

Syfte med intervjun: Upplevelser av vården och hur du önskar att den ska fungera

Hur går det till?

Berätta gärna fritt!

Inga svar är rätt eller fel det är dina erfarenheter och upplevelser och tankar det handlar om.

Jag spelar in - skriver ner intervjun. Intervjun enbart märkt med en sifferkod.

Kodlista som enbart jag och mina handledare har tillgång till.

Anonym sammanställning av resultatet. Ingen vet vad just du svarat.

Frivilligt!

Har du några frågor innan vi börjar intervjun?

Warming up talk

Vill du berätta lite om dig själv?

Vad gör du till vardags?

Vad tycker du om att göra på fritiden?

Vad har du för intressen?

Vill du berätta något mer om dig själv? Något du tycker är kul att göra

Allmänt om sjukhus och sjukhusvistelser

Har du varit mycket på sjukhus?

Vilka olika sjukhus har du varit på? Olika avdelningar?

Vet du hur det var när du föddes?

Vill du berätta vilka medicinska problem man upptäckte när du föddes?

Har du blivit opererad flera gånger?

Hur tycker du det känns att besöka ett sjukhus idag? Vilka känslor och tankar få du?

När du tänker tillbaka på alla gånger du varit på sjukhus:

Kommer du ihåg några speciella tillfällen? Vill du berätta lite om det?

Några speciella händelser? Vill du berätta lite om det?

Vad är det som har varit bra/positivt när du har varit på sjukhuset?

Vad är det som varit mindre bra eller dåligt/negativt på sjukhus?

Vad har du tyckt varit jobbigt eller obehagligt? Kan du berätta hur du upplevde det?

Berätta om när du känt dig rädd

Berätta om när du upplevt smärta

Hur tycker du att personalen har varit mot dig/ bemött dig

Vad har varit bra i kontakten med personalen? Vad har varit mindre bra?

Någon speciell händelse som du minns? Positivt eller negativt?

Om du försöker att tänka på alla tillfällen du varit på sjukhus vad känner du då?

Är det något mer du vill berätta om dina upplevelser på sjukhus?

Nuläge

Hur mycket kontakt har du med sjukvården nu för tiden?

Nu är ju du vuxen och jag antar att du inte får fortsätta komma på kontroller inom barnsjukvård. Har du kontakt med vuxensjukvården nu?

Vilken typ av mottagning, avdelning? (vuxen eller barn?)

Vad är det som gör att behöver du komma till sjukhuset?

Vill du berätta vilka typer av kontroller du går på?

Om du **inte** har en regelbunden kontakt med vuxensjukvården - vet du vart du ska vända dig om du behöver kontakta sjukvården?

Två alternativa fortsättningar av intervjun:

1. Överförd till vuxensjukvård

Erfarenheter av överföring överflyttning alternativt att få lämna barnsjukvården

Hur länge fick du gå på kontroller i barnsjukvården?

När flyttades du över till vuxensjukvården?

Kommer du ihåg vad du tänkte inför överflyttningen?

Kommer du ihåg vilka förväntningar du hade för överflyttningen?

Kommer du ihåg om du var orolig för något?

Förberedelse

Kommer du ihåg hur det förbereddes?

Fick du information i förväg? Långt i förväg? Hur fick du information? När?

Vilken typ av information?

Vem var det som var inblandad i processen?

Fick du träffa vuxenpersonalen innan du överflyttades helt? Berätta mer om det.

Erfarenheter av processen

Vad tänker du så här efteråt om överflyttningen?

Hur var det att byta klinik och personal?

Svårigheter? Nackdelar? Fördelar?

Om du jämför barnsjukvården och vuxensjukvården:

Vad är skillnaden mellan avdelningar och mottagningar för barn jämfört med för vuxna?

Vilka är likheterna?

Vad har blivit annorlunda i din kontakt med vården efter överflyttningen?

Hur tycker du det har det blivit?

Vad har blivit bättre? Vad har blivit sämre?

2. Ingen planerad fortsatt uppföljning

Hur tänker du om att det inte finns någon planerad uppföljning?

Vad sa dom på barnmottagningen när du avslutades?

Fick nu någon information från barnmottagningen om fortsatt uppföljning när du avslutades där?

Vet du vart du ska vända dig om du får problem i fortsättningen?

Tror du dina föräldrar vet det?

Hur önskar du att kontakten med sjukvården kommer att fungera i fortsättningen när du fyllt 18 år?

Gemensam fortsättning av intervjun 1 + 2

Praktiska kontakter

Hur kan du komma i kontakt med din avdelning eller mottagning när du behöver det?

Hur fungerar det att få kontakt?

Vem tar kontakt med vården när det behövs?

Följer dina föräldrar med? Är dina föräldrar involverade i vården?

Hur mycket är du med och bestämmer om din vård och behandling?

Vilka **önskemål h**ar du för fortsättningen i din kontakt med sjukvården?

Vilka råd kan du ge i samband när vi ska flytta över ungdomar till vuxensjukvården?

Förslag på hur man kan göra på ett bra sätt?

Är det något annat du vill ta upp och berätta om i samband med överflyttningen?

Sammanfattning hur jag uppfattat informationen i intervjun

Tack för att du delade med dig!

Får jag återkomma om jag har frågor?

Uppföljningsfrågor: Hur upplevde du det?
Hur menar du då?
Kan du beskriva…..?
Kan du berätta…….?
Hur kändes det då….?
Vad gjorde du då?
Kan du säga något mer om det?
