# Supplementary material for: Understanding of the transition to adult healthcare services among individuals with VACTERL association in Sweden: A qualitative study
Source: PLoS One. 2022 May 27;17(5):e0269163. doi: 10.1371/journal.pone.0269163 (PMC9140225; doi:10.1371/journal.pone.0269163)
Supplement: S4 File — (PDF) [file pone.0269163.s004.pdf]

S4 File. Interview guide for young adults and adults in English

Introductory questions/information

Presentation of myself, why I'm interested.

Purpose of the interview: Experiences of health care and how you would like it to function

How does it work?

Feel free to speak frankly!

There are no right or wrong answers, it is your experiences, understanding and thoughts that this is about.

I record - transcribe the interview. The interview is only marked with a number code.

A code list that only I and my supervisors have access to.

Anonymous compilation of the results. Nobody knows what specifically you have answered.

Voluntary!

Do you have any questions before we start the interview?

Warming up talk

Would you like to tell me a little about yourself?

What do you do in your daily life?

What do you like to do in your spare time?

What interests do you have?

Would you like to tell me something more about yourself? Something you think it's fun to do

General information about hospitals and hospital stays

Have you been to hospital a lot?

What different hospitals have you been to? What different wards?

Do you know how it was when you were born?

Would you like to tell me about what medical problems/issues that were discovered when you were born?

Have you been operated on several times?

How does it feel to visit a hospital today? What are your feelings and thoughts?

When you think back on all the times you've been in hospital:

Do you remember any special occasions? Can you tell me more about them?

Any special events? Can you tell me more about them?

What has been good about when you've been in hospital?

What has not been so good or even bad in hospital?

What have you felt has been difficult or unpleasant? Can you tell me how you experienced that?

Tell me about when you have felt scared

Tell me about when you experienced pain

How do you feel the staff has been towards you?

What has been good about your contact with the staff? What hasn't been so good?

Any special event you remember? Good or bad?

If you try to think of all the times you've been to hospital, how does that make you feel?

Is there anything else you want to tell us about your hospital experiences?

Current situation

How much contact do you have with health care nowadays?

Now when you are an adult, I don't suppose you continue to come for check-ups within pediatric care. Are you in contact with adult health care now?

What type of clinic, ward? (adult or pediatric)

Why do you need to come to hospital?

Would you like to tell me about what kinds of check-ups you come for?

If you **don't** have regular contact with the adult healthcare system – do you know where to turn if you should need to contact health care?

Two alternative continuations for the interview:

1. Transferred to adult health care

Experience of transferring/shifting alternatively leaving pediatric care

How long did you go for check-ups within pediatric care?

When were you transferred to adult health care?

Do you remember your thoughts prior to the transfer?

Do you remember what expectations you had regarding the transfer?

Do you remember if you were worried about anything?

Preparation

Do you remember how it was prepared?

Did you get information in advance? Far in advance? How did you get the information? When?

What kind of information?

Who was involved in the process?

Did you get to meet the adult staff before your transfer was completed? Tell me more about that.

Experiences of the process

What are your thoughts now after the transfer?

What was it like to change clinics and staff?

Difficulties? Disadvantages? Benefits?

If you compare pediatric health care and adult health care:

What is the difference between wards and clinics for children compared to those for adults?

What are the similarities?

What is different in your contact with health care after the transfer?

How do you think it has become?

What has got better? What has got worse?

2. No planned further follow-up

How do you feel about the fact that there is no planned follow-up?

What did they say at the pediatric clinic when you were finished?

Did you receive any information from the pediatric clinic about further follow-up when you were finished there?

Do you know where to turn if you have problems in the future?

Do you think your parents know?

How would you like your contact with the healthcare system to work in the future when you've turned 18?

Common continuation of the interviews 1 + 2

Practical contacts

How can you get in touch with your ward or clinic when you need to?

How does getting in touch work?

Who contacts the healthcare services when necessary?

Do your parents come with you? Are your parents involved in your care?

How much are you involved in deciding on your care?

What **wishes** do you have for the continuation of your contact with health care?

What advice would you like to give in connection with when we transfer young people to adult care?

Suggestions on how to do this in a good way?

Is there anything else you want to bring up and talk about in conjunction with the transfer?

Summary of how I perceived the information in the interview

Thank you for sharing this with me!

May I get back to you if I have any questions?

Follow-up questions:

How did you experience it?

How do you mean?

Can you describe…..?

Can you tell me about …….?

How did it feel then….?

What did you do then?

Can you tell me something more about that?
