# Supplementary material for: Understanding of the transition to adult healthcare services among individuals with VACTERL association in Sweden: A qualitative study
Source: PLoS One. 2022 May 27;17(5):e0269163. doi: 10.1371/journal.pone.0269163 (PMC9140225; doi:10.1371/journal.pone.0269163)
Supplement: S5 File — (PDF) [file pone.0269163.s005.pdf]

S5 File. COREQ checklist.

Consolidated criteria for reporting qualitative studies (COREQ): 32-item checklist

No	Item	Guide questions/description
**Domain 1: Research team and reflexivity**		
Personal Characteristics		
1.	Interviewer/facilitator	Which author/s conducted the interview or focus group?<br><br>*The first author (A-MK) conducted the interviews, reported in Material and Methods.*
2.	Credentials	What were the researcher's credentials?<br><br>*1:st author: PhD,*<br>*2:nd author: PhD, Assistant professor,*<br>*3:rd author PhD,*<br>*4th author: PhD, MD, professor*<br>*See the affiliations.*
3.	Occupation	What was their occupation at the time of the study?<br><br>***1:st author:** RN, Pediatric nurse, research nurse.*<br>***2:nd author:** Assistant professor, Researcher, RN, Pediatric nurse.*<br>***3:rd author:** Senior Pediatric Nurse Specialist, RN, MSc, PCNS.*<br>***4th author:** Professor, Consultant in Pediatric Surgery*

No	Item	Guide questions/description
4.	Gender	Was the researcher male or female?<br><br>*All authors female.*
5.	Experience and training	What experience or training did the researcher have?<br>*1:st author: More than 10 years' experience as in-patient pediatric nurse, trained as pediatric and research nurse, post-graduate courses in Interview techniques in health and care and in Qualitative content analysi*s.<br>*Short description included in Material and Methods; Method and data collection.*
Relationship with participants		
6.	Relationship established	Was a relationship established prior to study commencement?<br><br>*The first author was not involved in the regular care of these adolescents, young adults and adults. Relationship was established through telephone contact after an information letter was sent and consent obtained.*<br><br>*Reported in the Material and Methods; Method and data collection.*
7.	Participant knowledge of the interviewer	What did the participants know about the researcher?<br><br>*The invitation letter included information about the 1: st author being an experienced pediatric nurse involved in a PhD student project and later PhD,*

No	Item	Guide questions/description
		with the aim of investigating experiences in connection with transfer to adult health care. Procedure approved by the Regional Ethical Review Board in Uppsala.
8.	Interviewer characteristics	What characteristics were reported about the interviewer/facilitator?<br><br>*The interviewer was not involved in the regular care of this group of children, reported in Material and Methods and in Discussion; Methodological considerations.*
**Domain 2: study design**		
Theoretical framework		
9.	Methodological orientation and Theory	What methodological orientation was stated to underpin the study?<br><br>*Methodical orientation was Qualitative content analysis, reported in Material and Methods; Data Analysis.*
Participant selection		
10.	Sampling	How were participants selected?<br><br>*Purposive sampling was applied.*<br><br>*Adolescents, young adults and adults with the diagnosis VACTERL association aged 15 - 35 years treated in*

No	Item	Guide questions/description
		three Swedish pediatric surgical center were invited for interviews.<br><br>*Eighteen adolescents aged 15 -17 were invited until 10 were interviewed.*<br><br>*Among the young adults 16 were invited up to November 2020 whereof 8 agreed to be interviewed. The remaining eligible young adults were just turning 18 years and since only few of the previously interviewed 18 years old young adults had been transferred to adult health care we choose to invite 11 patients above 20 from one center. Four of these agreed to participate.*<br><br>*An invitation was also spread through the VACTERL national peer association by information in their website and in meetings.*<br><br>*Out of 45 approached persons 20 agreed to participate. Two additional persons was recruited through the peer association. Reported in the Material and Methods; Participants.*
11.	Method of approach	How were participants approached?<br><br>*The persons were invited by mail and then contacted by telephone to ask about their interest to participate. Through the peer association information was spread in their web site and in meetings, reported in the Material and Methods; Participants.*
12.	Sample size	How many participants were in the study?

No	Item	Guide questions/description
		10 adolescents, 8 young adults and 4 adults, reported in the Material and Methods; Participants.
13.	Non-participation	How many people refused to participate or dropped out? Reasons?<br><br>*Out of 45 approached persons 20 provided consent. Thus, 25 did not agree to participate for unknown reasons. Two additional persons contacted by themselves the researcher. Reported in the Material and Methods; Participants.*
Setting		
14.	Setting of data collection	Where was the data collected?<br><br>*The participants were interviewed by telephone in their homes, reported in the Material and Methods; Method and data collection.*
15.	Presence of non-participants	Was anyone else present besides the participants and researchers?<br><br>*In one interview the mother of the participant was present in the same room.*
16.	Description of sample	What are the important characteristics of the sample?<br><br>*The interviewed adolescents, young adults and adults were patients with the diagnosis of VACTERL association treated in three out of four pediatric surgical centers in Sweden. Interviews were performed between June 2016 and March 2021, Reported in the Material*

No	Item	Guide questions/description
		and Methods; Method and data collection.
Data collection		
17.	Interview guide	Were questions, prompts, guides provided by the authors? Was it pilot tested?<br><br>*Interview guide constructed in close cooperation with the supervisor for the PhD project. No pilot testing performed. Interview guide in original and English language added as Supporting information (S1 File, S2 File, S3 File, S4 File).*
18.	Repeat interviews	Were repeat interviews carried out? If yes, how many?<br><br>*No repeated interviews were carried out.*
19.	Audio/visual recording	Did the research use audio or visual recording to collect the data?<br><br>*Audio recording was performed, reported in the Material and Methods; Method and data collection.*
20.	Field notes	Were field notes made during and/or after the interview or focus group?<br><br>*Reflections after the interviews were written down, reported in the Material and Methods; Method and data collection.*

No	Item	Guide questions/description
21.	Duration	What was the duration of the interviews or focus group?<br><br>*Median length 33 (17-60) minutes; among adolescents 31.5 (23-42), young adults: 31 (17-39) and adults 49 (40-60) minutes, reported in the Material and Methods; Method and data collection.*
22.	Data saturation	Was data saturation discussed?<br><br>*Yes, data richness and saturation was included in the Discussion.*
23.	Transcripts returned	Were transcripts returned to participants for comment and/or correction?<br><br>*Transcripts were not returned.*
**Domain 3: analysis and findings**		
Data analysis		
24.	Number of data coders	How many data coders coded the data?<br><br>*The analysis was performed in collaboration between the authors until consensus was reached, reported in the Material and Methods; Data analysis and discussed in the Discussion.*
25.	Description of the coding tree	Did authors provide a description of the coding tree?<br><br>*The coding three is not presented in the study however, described in the text in the Material and Methods; Data*

No	Item	Guide questions/description
		analysis. A coding tree was used during the analysis by NVivo, reported in the Material and Methods; Data analysis.
26.	Derivation of themes	Were themes identified in advance or derived from the data?<br><br>*Themes were formulated from the collected data using inductive method, reported in the Material and Methods; Data analysis.*
27.	Software	What software, if applicable, was used to manage the data?<br><br>*The software NVivo for Windows, Release 1.4.1 software (QSR International Pty Ltd, Victoria, Australia) was used for organizing and visualizing the data, reported in the Material and Methods; Data analysis.*
28.	Participant checking	Did participants provide feedback on the findings?<br><br>*No participant checking was used.*
Reporting		
29.	Quotations presented	Were participant quotations presented to illustrate the themes / findings? Was each quotation identified?<br><br>*Quotations with participant number and and age group illustrate the categories, reported in Results.*
30.	Data and findings consistent	Was there consistency between the data presented and the findings?

No	Item	Guide questions/description
		Yes, see the Results and discussed in the Discussion.
31.	Clarity of major themes	Were major themes clearly presented in the findings?<br><br>*Yes, a table is displayed for Categories and Subcategories. An overarching theme was formulated catching the underling meaning based on the findings. Reported in Results.*
32.	Clarity of minor themes	Is there a description of diverse cases or discussion of minor themes?<br><br>*Yes, in the Results and in the Discussion. The finding based on the content is described in the Subcategories and quotation is added to support the labeling of the Subcategories in the Results.*
