# Supplementary material for: Understanding of the transition to adult healthcare services among individuals with VACTERL association in Sweden: A qualitative study
Source: PLoS One. 2022 May 27;17(5):e0269163. doi: 10.1371/journal.pone.0269163 (PMC9140225; doi:10.1371/journal.pone.0269163)
Supplement: S6 File — (PDF) [file pone.0269163.s006.pdf]

S6 File. Information for youngsters 15 - 17 years and request for participation in the study

"Experiences, expectations and wishes in conjunction with transfer to adult healthcare services among adolescents and young adults with a diagnosis of oesophageal atresia, anal atresia and VACTERL - an interview study"

Hi!

We are a group that works with something we call "Value-based care" at the Department of Paediatric Surgery, Akademiska barnsjukhuset (Uppsala University Children's Hospital). The goal is to find out what is important for children and youngsters and their parents in their contact with healthcare services and to find areas that can be better for them. We have focused on the group of patients diagnosed with oesophageal atresia, anal atresia and VACTERL and their families. To find out what is important for youngsters in contact with healthcare services and especially considering that you will sometime in the future been transferred from paediatric care to adult care, we would like to carry out an interview with you.

Request for participation

From the information we have at the University Hospital, we have found that you have had surgery for oesophageal atresia and/or anal atresia or have been diagnosed with VACTERL. We therefore ask you if you would like to participate in this interview study.

Why are we carrying out this study?

We want to investigate the perceptions and experiences of healthcare services among teenagers aged 15-17 with the diagnosis oesophageal atresia, anal atresia and VACTERL. By interviewing you, we want to find out what is important to you in your contact with healthcare services and what your expectations and wishes are with respect to adult care when it is time for you to transfer to this care form.

How is the study done?

I, who will be carrying out the interviews, am a paediatric nurse with many years of experience and am now working on a doctoral project to investigate experiences of

healthcare services among patients with the same diagnosis as you have. I will carry out the interviews in an undisturbed room when you come to the hospital for a visit or in your home or, if you wish, by phone. You can decide what is best for you. I will ask you a number of questions, but ideally, I would like you to speak as freely as possible about what you think about the health care you have experienced. No answer is right or wrong, I just want you to tell me how you have experienced it. If you want to talk about something negative, be assured that no one will find out that this information has come from you.
I will carry out the interviews in a place where no one else can hear what you are saying. You are welcome to ask your mother or father to be present at the interview, but we want only you to speak. The interview will be recorded on an MP3 player, then printed and compiled into a paper. About 10 young people will be asked to answer these questions and it is important that you know that no one will know what just you have said.

Are there any risks involved in participating?
We do not see that there are any risks involved in participating in the study. No painful or risky procedures are included. The recorded interviews will be handled so that no unauthorised person will know who answered or who said what.

Are there any benefits?
We cannot promise that the study will result in direct benefits to you. However, by getting viewpoints directly from you, the youngsters with the diagnosis oesophageal atresia, anal atresia and VACTERL, we hope that in the future there will be improvements in the provision of the healthcare services.

Data management and confidentiality.
All information from you and the interviews will be stored so that no unauthorised person can gain access to it. All personal data will be stored digitally and only handled by members of the research group.

How can I access the results of the study?

The results of the study will be made available when they are published in a scientific journal. Data will be reported at group level and it will not be possible to know what just you have said.

Voluntarism

Participation in this study is voluntary and you can withdraw your participation at any time, without giving any explanation and without this affecting your future treatment.

If we have not heard anything from you, we will call you in about two weeks to find out if you are interested or not in participating in an interview. In the case that you are interested, we can plan for a suitable time to do this.

If you can already now decide whether or not you want to be part of the study, please fill in the form at the end of this letter and send it back to us in the enclosed stamped addressed envelope.

Responsible for the study:

Ann-Marie Kassa
Pediatric nurse, PhD student
Pediatric Surgery Clinic
Uppsala University Children's Hospital, Uppsala
ann-marie.kassa@kbh.uu.se
076-2114259

Supervisors:

Helene Engstrand Lilja
Senior consultant, professor
Pediatric Surgery Clinic
Uppsala University Children's Hospital, Uppsala.
helene.lilja@kbh.uu.se

Gunn Engvall
Pediatric nurse, associate professor
Women's and children's health
Uppsala University
gunn.engvall@kbh.uu.se

I have received information about the study

"Experiences, expectations and wishes in conjunction with transfer to adult healthcare services among adolescents and young adults with a diagnosis of oesophageal atresia, anal atresia and VACTERL - an interview study"

Yes, *I want to participate in the interview study* ☐

No, *I don't want to participate in the interview study* ☐

Your personal identity number:_______________________________

Your name:____

____

Place and date

____

Signature
