# Supplementary material for: Understanding of the transition to adult healthcare services among individuals with VACTERL association in Sweden: A qualitative study
Source: PLoS One. 2022 May 27;17(5):e0269163. doi: 10.1371/journal.pone.0269163 (PMC9140225; doi:10.1371/journal.pone.0269163)
Supplement: S7 File — (PDF) [file pone.0269163.s007.pdf]

S7 File. Parental information and request for participation in the study **"Experiences, expectations and wishes in conjunction with transfer to adult healthcare services among adolescents and young adults with a diagnosis of oesophageal atresia, anal atresia and VACTERL - an interview study"**

Background

Work within "Value-based care" began in 2014 at the Department of Paediatric Surgery, Akademiska barnsjukhuset (Uppsala University Children's Hospital). This work aims to find areas in healthcare services that can be improved and create the greatest possible value for patients diagnosed with oesophageal atresia, anal atresia or VACTERL and their families. We want to conduct an interview study to find out what is important for young people in their contact with healthcare services, especially considering that these young people shall be transferred from paediatric care to adult health care.

Request for participation

From the patient administration system at the University Hospital, we have retrieved information that your child has had surgery for oesophageal atresia and/or anal atresia or has been diagnosed with VACTERL. We therefore ask you if you agree to your child participating in this study.

What is the purpose of the study?

The purpose is to investigate the perceptions and experiences of healthcare services among the group of teenagers aged 15-17 with the diagnosis oesophageal atresia, anal atresia and VACTERL. By interviewing them, we want to find out what is important to these teenagers in their contact with healthcare services and what their expectations and wishes are with respect to adult care when it is time for them to transfer to this care form.

How is the study done?

I, who will be carrying out the interviews, am a paediatric nurse with many years of experience and am now working on a doctoral project to, among other things, investigate this group's experiences of healthcare services. The interviews with the youngsters will be carried out in an undisturbed room when you come to the hospital

for a visit. It is also possible to carry out the interviews in your home if you wish or, what may be simpler, by phone. A number of questions will be used to aid the conversation, but the goal is that the youngsters speak as freely as possible about their experiences and wishes. The interviews will be conducted in an undisturbed room and in such a way that unauthorised persons will not gain access to the information. Parents are welcome to be present in the room, but we wish that only the youngsters participate in the interview. The interviews will be recorded on an MP3 player and will then be written down verbatim. An analysis of the content of the interviews will be made to describe the experiences. A total of about 10 young people in this age group are expected to be interviewed.

What are the risks?
We do not see that there are any risks involved in participating in the study. No painful or risky procedures are included. The recorded interviews will be handled so that no unauthorised person will know who has participated.

Are there any benefits?
We cannot promise that the study will result in direct benefits to you and your child. However, for the whole group of children and young people with oesophageal atresia, anal atresia and VACTERL, the experiences shared can in the future lead to improvements in the provision of the healthcare services.

Data management and confidentiality.
All data will be stored so that no unauthorised person can access it. All personal data will be digitally stored and only handled by members of the research group. Data will not be disclosed to companies or sent abroad. The Personal Data Act applies (1998: 204), which means that you can access registered information collected about your child once a year, and if necessary correct inaccurate information. You should then contact those responsible for the study, please see contact information below. The controller of personal data is the County Council in Uppsala County.

How can I access the results of the study?

The results of the study will be made available when they are published in a scientific journal. Data will be reported at group level and cannot be traced to an individual.

Voluntarism

Participation in this study is voluntary and one can withdraw one's participation at any time, without giving any explanation. If one chooses not to participate, this will not affect future treatment or follow up.

If we have not heard anything from you within two weeks, we will call you to ask if your teenager is interested or not in participating. Thereafter we can plan for a suitable time for an interview if interested.

If your teenager already knows whether or not s/he want to be part of the study, please fill in the form at the end of this letter and send it back to us in the enclosed stamped addressed envelope.

Responsible for the study:

Ann-Marie Kassa
Pediatric nurse, PhD student
Pediatric Surgery Clinic
Uppsala University Children's Hospital, Uppsala
ann-marie.kassa@kbh.uu.se
076-2114259

Supervisors:

Helene Engstrand Lilja
Senior consultant, professor
Pediatric Surgery Clinic
Uppsala University Children's Hospital, Uppsala.
helene.lilja@kbh.uu.se

Gunn Engvall
Pediatric nurse, associate professor
Women's and children's health
Uppsala University
gunn.engvall@kbh.uu.se

We have received information about the study

"Experiences, expectations and wishes in conjunction with transfer to adult healthcare services among adolescents and young adults with a diagnosis of oesophageal atresia, anal atresia and VACTERL - an interview study"

Yes*, our teenager wants to participate in the interview study* ☐

Yes*, our teenager does not want to participate in the interview study* ☐

The child's/youth's personal identity number:________________________________

The child's/youth's name:____

If our teenager has said yes, we give our consent to participation in the interview study:

______________________________	______________________________
Place and date	Place and date
______________________________	______________________________
Guardian's signature	Guardian's signature
______________________________	______________________________
Guardian's name in block letters	Guardian's name in block letters
