# Supplementary material for: Understanding of the transition to adult healthcare services among individuals with VACTERL association in Sweden: A qualitative study
Source: PLoS One. 2022 May 27;17(5):e0269163. doi: 10.1371/journal.pone.0269163 (PMC9140225; doi:10.1371/journal.pone.0269163)
Supplement: S8 File — (PDF) [file pone.0269163.s008.pdf]

S8 File. Information for young adults 18 - 35 years and request for participation in the study

"Experiences, expectations and wishes in conjunction with the transfer to adult care among adolescents and young adults with a diagnosis of oesophageal atresia, anal atresia and VACTERL - an interview study"

Hi!

We are a group that works with something we call "Value-based care" at the Department of Paediatric Surgery, Akademiska barnsjukhuset (Uppsala University Children's Hospital). The idea is to find out what is important for children and young people and their parents in their contact with healthcare services and to find areas that can be better for them. We have focused on the group of patients diagnosed with oesophageal atresia, anal atresia and VACTERL and their families. To find out what is important for young adults in contact with healthcare services and especially considering that you have recently been transferred from paediatric care to adult care, we would like to carry out an interview with you.

Request for participation

From the information we have at the University Hospital, we have found that you have had surgery for oesophageal atresia and/or anal atresia or have been diagnosed with VACTERL. We therefore ask you if you would like to participate in this interview study.

What is the purpose of the study?

We want to investigate the perceptions and experiences of healthcare services among young adults aged 18-35 with the diagnosis oesophageal atresia, anal atresia and VACTERL. By interviewing you, we want to find out what is important to you in your contact with healthcare services and how you have experienced the transfer from paediatric care to adult care. We also want to find out how you experience your contact with healthcare services today.

How is the study done?

I, who will be carrying out the interviews, am a paediatric nurse with many years of experience and am now working on a doctoral project to investigate experiences of healthcare services among patients with the same diagnosis as you have. I will carry out the interview with you either in an undisturbed room when you come for a visit to the hospital or by phone. You can choose which you think is best. I will have some questions which I will ask you, but ideally, I would like you to speak as freely as possible about what you think of the health care that you have experienced. No answer is right or wrong, I just want you to tell me how you have experienced it. If you want to talk about something negative, be assured that no one will find out that this information has come from you.

Are there any risks involved in participating?

We do not see that there are any risks involved in participating in the study. No painful or risky procedures are included. The recorded interviews will be handled so that no unauthorised person will know who answered or who said what.

Are there any benefits?

We cannot promise that the study will result in direct benefits to you. However, by getting viewpoints directly from you, young adults with the diagnosis oesophageal atresia, anal atresia and VACTERL, we hope that in the future there will be improvements in the provision of these healthcare services.

Data management and confidentiality.

All data will be stored so that no unauthorised person can access it. All personal data will be digitally stored and only handled by members of the research group. Data will not be disclosed to companies or sent abroad. The Personal Data Act applies (1998: 204), which means that you can access registered information collected about you once a year, and if necessary correct inaccurate information. You should then contact those responsible for the study, please see contact information below. The controller of personal data is the County Council in Uppsala County.

How can I access the results of the study?

The results of the study will be made available when they are published in a scientific journal. Data will be reported at group level and cannot be traced to an individual.

Voluntarism

Participation in this study is voluntary and you can withdraw your participation at any time, without giving any explanation. If you choose not to participate, this will not affect future treatment or follow up.

If we have not heard anything from you, we will call you in about two weeks to find out if you are interested or not in participating in an interview. In the case that you are interested, we can plan for a suitable time to do this.

If you can already now decide whether or not you want to be part of the study, please fill in the form at the end of this letter and send it back to us in the enclosed stamped addressed envelope.

Responsible for the study:

Ann-Marie Kassa
Pediatric nurse, PhD student
Pediatric Surgery Clinic
Uppsala University Children's Hospital, Uppsala
ann-marie.kassa@kbh.uu.se
076-2114259

Supervisors:

Helene Engstrand Lilja
Senior consultant, professor
Pediatric Surgery Clinic
Uppsala University Children's Hospital, Uppsala.
helene.lilja@kbh.uu.se

Gunn Engvall
Pediatric nurse, associate professor
Women's and children's health
Uppsala University
gunn.engvall@kbh.uu.se

I have received information about the study

"Experiences, expectations and wishes in conjunction with transfer to adult healthcare services among adolescents and young adults with a diagnosis of oesophageal atresia, anal atresia and VACTERL - an interview study"

Yes*, I want to participate in the interview study* ☐

No, *I don't want to participate in the interview study* ☐

Your personal identity number:_______________________________

Your name:____

____

Place and date

____

Signature
